# Sudden Death from Asphyxia in a Phthisical Boy

**Published:** 1884-09

**Authors:** W. Barrett Roué

**Affiliations:** Physician to the Bristol Hospital for Sick Children


### SUDDEN DEATH FROM ASPHYXIA IN A PHTHISICAL BOY. By W. Barrett Roué. M.D., M.S., F.R.A.S., Physician to the Bristol Hospital for Sick Children.

E. P. A., a wretched, neglected-looking boy, æt. 11, was admitted into the above hospital. The history of his case was that of a cold, resulting in some chest affection, for which he had had no medical advice. A constant cough and pain in the chest had been present for five months. On my first visit to the wards after the boy's admission, I found him sitting up in bed, breathing rather rapidly, but not seeming much distressed, and in the act of expectorating purulent sputum into a receptacle already nearly full.

On physical examination there appeared to be no flattening or falling in of the chest on either side, but the respiration was shallow. There was marked dulness at the right apex, both in front and behind, and large moist sounds were heard over the upper portion of the same lung, accompanied by small crepitations. There was increased vocal resonance all over the remainder of the right lung. On the left side there was exaggerated breathing, which I considered to be merely compensatory, with slightly prolonged expiration. Otherwise the lung seemed healthy.

The boy was treated for his cough, and an attempt was made to improve his general condition by the administration of cod-liver oil, milk, and beef-tea. In spite of this treatment, he steadily decreased in weight, as the following will show:—May 3rd, weight 51½ lbs.; 10th, 50 lbs.; 17th, 48 lbs. The temperature varied from 100° to 104°, usually highest at night-time. From these symptoms I considered the prognosis to be unfavourable, but did not by any means apprehend a very rapid or sudden termination to the case.

On May 19th, whilst sitting up in bed, and without any severe cough, blood mixed with air began to well from his nose and mouth, and in about three or four minutes he was dead, as it appeared, simply from asphyxia, the trachea and the bronchi being full of blood, which the boy was unable to expel.

*Post-mortem Examination.*—Body much emaciated; face mottled blue and red. Heart healthy; right side gorged with thick black blood, left side empty. The right lung was solidified throughout, except a small portion of the extreme apex, and composed of degenerated cheesy-looking material. There was a large irregular cavity, about the size of a goose's egg, or larger, in the lower part of the upper lobe, extending for a considerable distance into the lobe beneath. This cavity was situated more toward the posterior than the anterior aspect of the chest; indeed, there was scarcely any lung-tissue left beneath the pleura in the former situation. The cavity was full of soft, recent blood-clot, which passed into the communicating bronchus, and upwards towards the trachea. On removing the blood-clot, an artery about the size of a crow-quill was found severed, and the ends hanging down against the walls of the cavity, one on either side. This vessel, previous to its rupture, had evidently passed right across the cavity. The left lung was emphysematous, the air-cells and bronchial tubes were full of blood, and a clot passed a considerable distance up the left large bronchus; otherwise the lung was healthy.

*Remarks.*—Up to the moment of the occurrence of the hæmorrhage I think we have a fairly clear case of a condition which, for want of a better name, I will call "pneumonic phthisis." The little patient had probably suffered from an attack of pneumonia in the right lung, the products of which failing to be absorbed, had broken down, and then gradual disintegration had caused the cavity. The important question is, What was the immediate cause of death? Two causes only, I think, are worthy of consideration; viz., syncope from loss of blood, and asphyxia. There is certainly a third possible cause in shock; but the difficulties of arriving at any reliable conclusions as to this condition in children, when the physical signs of organic mischief are so decided as in this case, justify the little notice I have taken of shock as an agent of death. With regard, then, to syncope from loss of blood, the post-mortem evidence is certainly against it. In the first place, the condition of the body strikes us: the face was mottled and of a purple colour; in death from syncope it is usually very pale. In the second place, the right side of the heart was gorged with blood; in death from syncope all the cavities are usually empty. Really the patient lost very little blood, considering the rapidity of his death. Upon consideration, then, asphyxia seems to be the immediate cause of death suggested by the post-mortem examination, for the following reasons:—

1st. The right side of the heart was gorged, while the left was empty.

2nd. The large and smaller bronchi in both lungs, and even the air-cells in the sound lung, were filled with blood, so as effectually to cut off all communication between the air and the air-cells.

3rd. The face bore the mottled and purple hue so well known in cases of death from suffocation.

